# No Association Between Maternal Post-partum Depression and Vaccination Uptake of Infants: A Matched Cohort Study in a Large Health Maintenance Organization Database in Israel

**DOI:** 10.3389/fped.2021.771089

**Published:** 2022-02-08

**Authors:** Ariela Zaikin, Gideon Koren, Gabriel Chodick, Zachi Grossman

**Affiliations:** ^1^Department of Clinical Pharmacy, Faculty of Medicine, School of Pharmacy, Hebrew University of Jerusalem, Jerusalem, Israel; ^2^Department of Pharmacy, Schneider Children's Medical Center of Israel, Petah Tikva, Israel; ^3^Adelson School of Medicine, Ariel University, Ariel, Israel; ^4^Maccabitech Institute of Research and Innovation, Maccabi Healthcare Services, Tel-Aviv, Israel; ^5^Sackler Faculty of Medicine, Tel-Aviv University, Tel-Aviv, Israel; ^6^MaccabiTech, Maccabi Healthcare Services, Tel-Aviv, Israel

**Keywords:** antidepressants, Edinburgh Postnatal Depression Scale, primary care, child vaccination, maternal post-partum depression

## Abstract

**Background:**

Maternal post-partum depression is one of the most common medical complications around childbirth. One of its consequences is a possible association with children's receipt of preventive health care, including immunization. This study aimed to explore the association between maternal postpartum depression and children's immunization rates.

**Methods:**

A retrospective cohort study of Maccabi Healthcare Services (HMO) members comparing immunization rates between children born between 2006 and 2019 to mothers with post-partum depression and children born to mothers with no documented depression. Post-partum depression was assessed by Edinburgh Postnatal Depression Scale, a 10-item questionnaire considered the screening tool of choice in Israel for early Post-partum detection. 1:1 matching was conducted according to criteria: age of the mother ± 2 years, child's year of birth, the newborn baby's gender, the baby's birth order and socioeconomic index. The primary outcome was defined as non-vaccination and the exposure was defined as depression. A sub-analysis was performed, comparing immunization rates of children born to mothers treated with antidepressant medications to rates of children born to untreated mothers.

**Results:**

A total of 709 subjects in the exposed cohort (children born to mothers with post-partum depression symptoms) and 681 subjects in the matched cohort were analyzed. The relative risks among children born to depressed mothers compared with children born to healthy mothers for not receiving any vaccine at 2 months, three doses of pertussis vaccine up to 7 months and four doses of DTaP-Hib-IPV vaccine up to 18 months were 1.15 (95% CI 0.74–1.78), 1.11 (95% CI 0.94–1.31) and 0.82 (95% CI 0.56–1.95), respectively. The relative risks among 139 infants born to treated mothers compared with 570 infants born to untreated mothers for not receiving any vaccine at 2 months, three doses of pertussis vaccine up to 7 months and four doses of DTaP-Hib-IPV vaccine up to 18 months were 1.28 (0.64–2.54), 0.78 (0.57–1.06) and 0.42 (0.17–1.03), respectively.

**Conclusion:**

We found no significant association between maternal post-partum depression and uptake of child Immunization.

## Introduction

Maternal Post-Partum Depression (PPD), which occurs in the first 12 months after birth (1), is one of the common medical complications associated with childbirth (2, 3). The estimated prevalence of PPD is uncertain (4). The range is very wide, varying between 0.1 and 82.1%, depending on the methodology and characteristics of each study (5). According to a recently published systematic survey and meta-analysis that included 291 studies in 56 countries, the prevalence of PPD detected with the Edinburgh Postnatal Depression Scale (EPDS) is 17.7%. This survey included studies in Israel, where the prevalence varied from 5.2 to 43% (6).

Several tools have been developed for detecting PPD. EPDS is frequently employed in defining depression in both studies and clinical practice (2, 7). This questionnaire is the screening tool of choice in Israel for early PPD detection. It has been validated in Israel, and according to the Ministry of Health procedure for detecting women at risk of PPD, should be given twice: (1). To each pregnant woman starting in the 26th week of pregnancy, and (2). To each woman 4–9 weeks after birth, or on the following visit (8).

It has been found that PPD has negative effects on the mother-child relationship and the child's behavior and development (3, 9). One possible negative effect of PPD on the newborn's health involves obtaining regular treatment at the mother and child clinic during the early years of the child's life (10, 11). In general, in Israel fathers are less involved in various aspects of taking care of children compared to mothers (12–14). For that reason, we decided to focus in this study on maternal postpartum depression.

Vaccinations are regarded as a medical breakthrough that prevents 2–3 million deaths a year from diseases such as diphtheria, tetanus, pertussis, influenza, and measles (15). According to a report by the World Health Organization, the rate of vaccination coverage in Israel in 2018 varied from 81 to 99%, depending on the vaccine (16). In Israel, regular vaccines are administered free of charge at mother and child clinics and schools (17, 18). In order to determine the proper vaccination recommendations for babies and children, the Ministry of Health has developed quality indices for vaccination of babies and children at mother and child clinics in Israel. Two of them refer to the proportion of (19):

(i). Babies who reached 18 months of age and received four doses of the quintuple vaccination [Diphtheria-Tetanus-Acellular Pertussis Vaccine pediatric (DTaP)+ Inactivated Polio Vaccine (IPV)+ Haemophilus influenza b Vaccine (Hib)] by age 18 months.

(ii). Babies aged seven months and received three doses of a vaccine against pertussis.

The primary purpose of the study is to test whether PPD has a negative effect on the proportion of vaccination among children, while examining the vaccination patterns for babies born to mothers in whom PPD symptoms were detected according to EPDS. A secondary purpose is to examine whether the beginning of medication treatment for depression has an effect on the proportion of vaccination among children of mothers in whom PPD symptoms were detected according to EPDS.

Optimal response to childhood vaccines requires an understanding of family factors. The insights gained in this study will enhance the capabilities to increase this responsiveness.

## Materials and Methods

### Study Population and Database

This retrospective cohort study included children of all Maccabi Healthcare Services members who gave birth in the period between September 5, 2006 and February 23, 2019, and whose mothers filled out the EPDS questionnaire after birth. As of 2017, 25.4% of all Israeli health fund members were members of Maccabi Healthcare Services (20). The database contained the ID numbers of the mother and her children, the birth date of each child, the child's gender, the mother's birth date, a socioeconomic index (according to the Central Bureau of Statistics' geographic characterization code), the score on and date of filling out an EPDS questionnaire (including details of Question No. 10), and a list of vaccinations received by the children and the dates on which they were administered. The data was transferred to Maccabi Healthcare Services from “Tipat Halav”, a center that provides health and medical services in the field of health promotion and prevention for pregnant women, infants and children (from birth to age 6 years) and their families. Tipat Halav centers are distributed throughout Israel and are operated by the health bureaus (21).

### Classification of Postnatal Depression

PPD was defined as the exposure. After encoding, the scores on the EPDS questionnaire ranges from 0 to 30, with a higher score on the questionnaire indicating a greater risk of depression. A score of nine or less indicates a low risk of PPD, a score of 10 or higher a medium-to-high risk, and a positive response to Question No. 10 (“In the past week, the thought of harming myself has occurred to me”) indicates a high risk, with concern about self-harm (7, 8, 22).

Women with an EPDS score of 10 or higher and those answering yes to Question No. 10 (“Yes, quite often” or “Sometimes”) were therefore classified in the group of mothers with PPD symptoms. Women with an EPDS score of <10 were classified in the group of mothers with no PPD symptoms.

The secondary outcome was the immunization rates (defined as not receiving the vaccinations) of children born to mothers treated with antidepressant medications. A list of patients for whom treatment with antidepressants of any type had been initiated as a result of a response to an EPDS questionnaire was retrieved. The drugs included in the list were drugs that contained one of the active ingredients detailed in the attached Appendices.

### Infant Vaccinations

The primary outcome was defined as not receiving vaccinations.

The vaccination rates of the children in the two groups were compared according to the following indices:

#### Ministry of Health Quality Indices

(i). The proportion of babies over seven months old who received three doses of vaccine against pertussis, including: DTap, Diphtheria-Tetanus-Pertussis (DTP), DTAP+IPV, DTAP+ enhanced Inactivated Polio Vaccine (eIPV), DTaP+HIB, DTaP+HIB+IPV+ Hepatitis B Vaccine (HBV).

(ii). The proportion of babies who reached the age of 18 months during the measured period and who received four doses containing all parts of the quintuple vaccine (DTaP +IPV+Hib or DTP+IPV+Hib) before reaching the age of 18 months.

#### Additional Indices

(iii). The proportion of babies about 2 months old (from age 42 days, the minimum age for vaccination, up to age 83 days) who received a dose of at least one of the following vaccinations: IPV, DTaP (or DTP or DTAP), Hib, Pneumococcal Conjugate Vaccine (PCV13), Rotavirus Vaccine (Rota).

(iv). The proportion of babies about one-month-old (from age 28 days, the minimum age for vaccination, up to age 55 days) who received a dose of vaccine against HBV.

PPD is usually defined as depression in the first 12 months after giving birth (1). For this reason, the Ministry of Health quality indices that include vaccine doses given soon after birth were selected, without including the index of the proportion of babies who reached the age of 13 months and who received one dose of triple vaccine (MMR) or quadruple vaccine (MMRV). Indices iii and iv, which concern vaccinations on a date soon after the birth, were also added.

These four indices were also compared in a sub-analysis of the group of children whose mothers had PPD symptoms and were treated with antidepressants, and the group of children whose mothers had PPD symptoms but were not treated with antidepressants.

### Potential Confounders

Potential confounding may be the age of the mother, socioeconomic status of the mother and child, the child's gender, the baby's birth order and child's year of birth, since the potential change in attitude toward vaccination may influence the results.

### Statistical Analysis

Information containing demographic and socioeconomic particulars of the mothers was used in order to match mothers in the two groups according to criteria: age of the mother ± 2 years, child's year of birth, the newborn baby's gender, the baby's birth order (for example, matching an oldest child with another oldest child) and socioeconomic index. The socioeconomic index was based on characterization and classification of geographical units by the socio-economic level of the population, coded by Israel's Central Bureau of Statistics. This index is based on database from National Insurance Institute, the Ministry of Finance, the Ministry of Defense, the Ministry of Education, the Ministry of Transport and Road Safety, and the Population and Immigration Authority. The coding was based on multivariate analysis of demographic, social and economic characteristics calculated from these administrative sources for the population residing in the geographical units (23).

**Figure 1:**
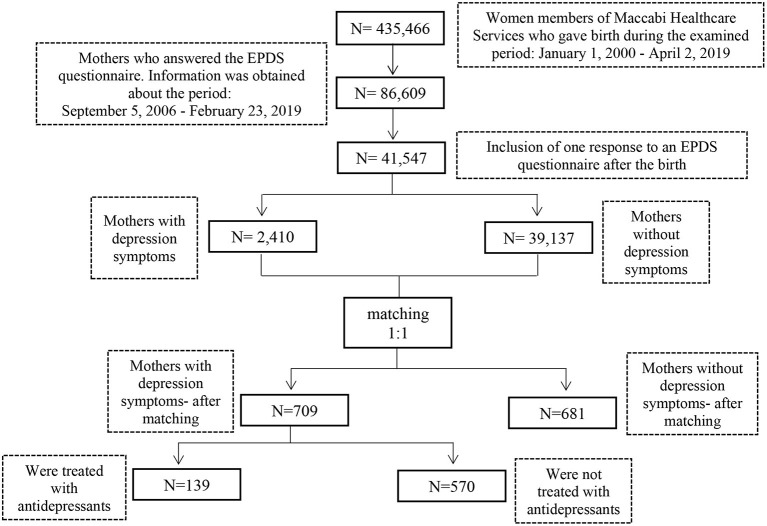
Flow chart depicting the inclusion process of the study cohort.

After matching by 1:1 according to criteria listed above, a sample size of *n* = 1,390 was obtained. A flow chart depicting the inclusion process of the study cohort is presented in Figure 1. The characteristics of the children whose mothers were treated with antidepressants were compared with those of the children whose mothers were not treated with antidepressants, using the chi-square test for categorical variables (gender of the infant and gestation time), the *t*-test for continuous variables (the mother's age), and the Mann-Whitney test for ordinal variables (birth order of the infant). Relative risk (RR) was calculated for each result with 95% Confidence Interval (CI) and a *p*-value (24). Missing data regarding EPDS scores, vaccinations of the children and socioeconomic particulars of the mothers was omitted. Samples with invalid data were not included in the matching and in further analysis. Information processing and statistical analysis were conducted in the Python programming language with the Pandas, NumPy, and Statsmodels packages, using the PyCharm platform (25–27).

### Ethics Requirements

The study was approved by the ethics review committees of The Tel Aviv Sourasky Medical Center Institutional Review Board (Helsinki Committee).

## Results

### Characteristics of the Children in the Cohort

According to the EPDS scoring, 5.8% of the 41,547 mothers suffered from PPD symptoms. Of the mothers, 2.4% had scores of over 13 in an EPDS questionnaire and were therefore identified as suffering from a major depressive disorder; 0.5% of the mothers who suffered from PPD symptoms answered a positive answer to Question No. 10, that is, identified as having self-harm ideation.

The characteristics of the group of children in the sample whose mothers had PPD symptoms and the group of children in the sample whose mothers had no PPD symptoms are displayed in Table 1. (In certain cases, the same patient with no PPD symptoms was used as a control for two mothers with PPD symptoms and the same matching criteria. N is therefore not identical for the two groups).

**Table 1:** Characteristics of the group of children whose mothers had postpartum depression symptoms and the group of children whose mothers had no postpartum depression symptoms.

**Variable**	**Children of mothers without depression symptoms (*N* = 681)**	**Children of mothers with depression symptoms (*N* = 709)**
Age of the mother (in years) on the date of birth^*^	31.5 ± 4.7	31.6 ± 4.7
**Year of the child's birth** ***N*** **(%)**		
2014>	67 (9.8%)	67 (9.5%)
2014–2016	377 (55.4%)	393 (55.4%)
2017–2019	237 (34.8%)	249 (35.1%)
Male children *N* (%)	375 (55.1%)	389 (54.9%)
Female children *N* (%)	306 (44.9%)	320 (45.1%)
Child's birth order^¥^	2	2

* *Average*

¥ *Median*.

The characteristics of the group of children in the sample whose mothers had PPD symptoms and who were treated with antidepressants and the group of children in the sample whose mothers had PPD symptoms but were not treated with antidepressants are displayed in Table 2.

**Table 2:** Characteristics of the group of children whose mothers had postpartum depression symptoms and who received drug treatment, and of the group of children whose mothers had postpartum depression symptoms and who did not receive drug treatment.

**Variable**	**Children with mothers not treated with antidepressants (*N* = 570)**	**Children with mothers treated with antidepressants (*N* = 139)**	***P*-value**
Age of the mother (in years) on the date of birth^*^	31.5 ± 4.8	32.0 ± 4.4	0.271
**Year of the child's birth** ***N*** **(%)**			
2014>	53 (9.3%)	14 (10.1%)	0.215
2014–2016	308 (54%)	85 (61.1%)	
2017–2019	209 (36.7%)	40 (28.8%)	
Male children *N* (%)	314 (55.1%)	75 (54.0%)	0.884
Female children *N* (%)	256 (44.9%)	64 (46.0%)	
Child's birth order^¥^	2	2	0.413

* *Average*

¥ *Median*.

### Receiving Vaccinations

The relative risk values of non-vaccination for children whose mothers had PPD symptoms in comparison with children whose mothers had no PPD symptoms are displayed in Table 3. It was found that the relative risk of children whose mothers had PPD symptoms in comparison with mothers who had no PPD symptoms was 0.98 (*p* = 0.919, 95% CI 0.68–1.44) for not being vaccinated with an HBV dose at age 1 month and 1.15 (*p* = 0.523, 95% CI 0.74–1.78) for not being vaccinated at age 2 months with at least one of the following vaccines: DTP/ DTAP/ DTaP, PCV13, Rota, Hib, IPV. In addition, the relative risk of children whose mothers had PPD symptoms in comparison with children of mothers having no PPD symptoms was 1.11 (*p* = 0.229, 95% CI 0.94–1.31) for not receiving three doses of vaccine against pertussis by age seven months and 0.82 (*p* = 0.299, 95% CI 0.56–1.95) for not receiving four doses of the quintuple vaccine (IPV+Hib+DTap/DTP) by age 18 months. According to our findings, no statistically significant association was found between the existence of PPD symptoms in mothers and the vaccination of their children.

**Table 3:** Relative risk values for non-vaccination of children whose mothers had PPD symptoms in comparison to children whose mothers had no PPD symptoms.

**Variable**	**Children of mothers without depression symptoms (not exposed) (*N* = 681)**	**Children of mothers with depression symptoms (exposed) (*N* = 709)**	**Relative risk for not receiving the vaccinations (95% CI)**	***P*-value**
***N*** **(%)**				
i. A dose of vaccine against HBV at age 1 month	633 (92.95%)	660 (93.09%)	0.98 (0.68–1.44)	0.919
ii. Vaccination at age 2 months	646 (94.86%)	667 (94.08%)	1.15 (0.74–1.78)	0.523
iii. Three doses of vaccine against pertussis by age seven months	500 (73.42%)	500 (70.52%)	1.11 (0.94–1.31)	0.229
iv. Four doses of the quintuple vaccine by age 18 months	627 (92.07%)	663 (93.51%)	0.82 (0.56–1.95)	0.299

*i. The proportion of babies aged 1 month who received a dose of vaccine against HBV. ii. The proportion of babies aged 2 months who received a dose of at least one of the following vaccines: IPV, DTaP / DTP / DTAP, Hib PVC13, Rota. iii. The proportion of babies over seven months old who received three doses of vaccine against pertussis. iv. The proportion of babies who received four doses of all parts of the quintuple vaccine (DTaP / DTP + IPV + Hib) by age 18 months*.

### Effect of Antidepressants on the Probability of Vaccination

The children whose mothers had PPD symptoms (*n* = 709) were divided into two groups: children with mothers who were not treated with antidepressants after filling up the EPDS questionnaire (*n* = 570) and children with mothers who were treated with any antidepressant whatsoever after filling out the EPDS questionnaire (*n* = 139). The relative risk of non-vaccination for these two groups is displayed in Table 4. The relative risk of children whose mothers who were treated with antidepressants in comparison with children whose mothers were not treated with antidepressants was 1.05 (*p* = 0.883, 95% CI 0.54–2.05) for not being vaccinated with an HBV dose at age 1 month and 1.28 (*p* = 0.478, 95% CI 0.64–2.54) for not being vaccinated at age 2 months with at least one of the following vaccinations: DTP/ DTAP/ DTaP, PCV13, Rota, Hib, IPV. It, therefore, follows that treatment with antidepressants for mothers with PPD symptoms does not affect the proportion of vaccination of their children at age 1 month and age 2 months. At the same time, the relative risk of children with mothers treated with antidepressants in comparison with children with mothers who were not treated with antidepressants was 0.78 (*p* = 0.116, 95% CI 0.57–1.06) for not receiving a vaccination against pertussis by age seven months and 0.42 (*p* = 0.058, 95% CI 0.17–1.03) for not receiving four doses of the quintuple vaccine (IPV + Hib + DTap / DTP) by age 18 months. For the later indices – up to age seven months and up to age 18 months – a trend is visible, albeit not a significant one, of a lower risk of non-vaccination of children with mothers being treated with antidepressants.

**Table 4:** Relative risk values for non-vaccination of children whose mothers had pospartum depression symptoms who were treated with antidepressants in comparison with children whose mothers had postpartum depression symptoms who were not treated with antidepressants.

**Variable**	**Children with mothers not treated with antidepressants (not exposed) (*N* = 570)**	**Children with mothers treated with antidepressants (exposed) (*N* = 139)**	**Relative risk for not receiving the vaccinations (95% CI)**	***P*-value**
***N*** **(%)**				
i. A dose of vaccine against HBV at age 1 month	531 (93.16%)	129 (92.81%)	1.05 (0.54–2.05)	0.883
ii. Vaccination at age 2 months	538 (94.39%)	129 (92.81%)	1.28 (0.64–2.54)	0.478
iii. Three doses of vaccine against pertussis by age seven months	386 (67.72%)	104 (74.82%)	0.78 (0.57–1.06)	0.116
iv. Four doses of the quintuple vaccine by age 18 months	521 (91.40%)	134 (96.40%)	0.42 (0.17–1.03)	0.058

*i. The proportion of babies aged 1 month who received a dose of vaccine against HBV. ii. The proportion of babies aged 2 months who received a dose of at least one of the following vaccines: IPV, DTaP / DTP / DTAP, Hib, PCV13, Rota. iii. The proportion of babies over seven months old who received three doses of vaccine against pertussis. iv. The proportion of infants over 18 months old who received four doses of all parts of the quintuple vaccine (DTaP / DTP + IPV + Hib) by age 18 months*.

## Discussion

We conducted a retrospective cohort study comparing immunization rates between children born between 2006 and 2019 to mothers with post-partum depression symptoms and children born to mothers with no documented depression symptoms. In our study, for the four variables tested, no association was found between the appearance of PPD symptoms in the mother and whether her children received regular vaccinations. However, administering antidepressant medication to depressed mothers may have a near association for a protective effect against not receiving childhood routine immunizations.

There are differing views in the literature regarding the association between maternal PPD and vaccination rate of their children. In a study by Turner et al. (11), an association was observed between the symptoms of depression or anxiety in the mother and regular vaccinations of children being postponed or not taking place at all. Another study examined the influence of maternal mental illness on vaccination uptake in children (28). This study focused on events of maternal depression, anxiety, psychosis, eating disorder, personality disorder and alcohol and substance misuse disorders, and adherence to DTaP/IPV/Hib and first dose MMR vaccines. The study demonstrated that children with maternal mental illness are significantly less likely to receive preventative health vaccinations during the first 5 years of life.

As demonstrated in our study, our results are in accordance with conflicting studies on the topic. Ruohomaki et al. investigated in a prospective cohort study the impact of postpartum depressive symptoms on self-reported infant health and analgesic consumption at the age of 12 months. 969 women during 2012–2017 were included in the investigation. No differences were detected between the postpartum elevated depressive symptoms and non-elevated depressive symptoms groups regarding adherence to regular vaccinations (29). In another prospective study, of Minkovitz et al. (9), which examined the relation between PPD in the mother and her children receiving preventative medical services in 5,565 families, only in the case of the MMR vaccine was a lower acceptance of routine vaccinations observed among children of mothers with PPD symptoms than in the group of children whose mothers had no PPD symptoms. For vaccination against DTP, which involves the period soon after the birth, no difference in vaccination rates was observed. In yet another study, Lyngsøe et al. (10), which focused on this subject, the RR in all the indices tested was close to one. They found a significant effect of depression, but of low magnitude. This study tested, in addition to preventative medical visits, vaccination rates against MMR and DiTe (diptheria /tetanus/ pertussis).

Our study has several advantages over the studies reported in the literature. First, those studies, except the study of Ruohomaki et al., did not use an EPDS questionnaire to define depression in mothers, which is a validated questionnaire used extensively in studies and clinical practice (2). Second, we focused on the first year after the birth, in which PPD is more relevant, and we included a variety of effects referring to different points in time subsequent to the birth, as well as a larger number of effects overall. Furthermore, we relied on data from the mother and child clinics to determine immunization rates and did not rely on maternal self-report. In addition, we used matching according to a number of variables between the children and the mothers in the two groups in order to obtain two groups with the most similar characteristics possible.

Another innovative advantage in this study is that it addresses treatment with antidepressants given to women in whom PPD was detected and examines its association on the vaccination rates of their children. There is information in the literature about the effect of treatment with antidepressants on the mother's functioning. In a study by Logsdon et al. (30), secondary data analysis for a double-blind random clinical trial, various functional and depression indices were assessed in 61 mothers diagnosed with depression before the beginning of treatment with antidepressants and after 8 weeks of treatment. The depression symptoms and most of the functional indices improved after the drug treatment was received. The same was true of another study by the same researchers, which demonstrated a trend of improvement in general functioning and maternal functioning in women with PPD who received short-term treatment with antidepressants (31).

In our study, in the results involving vaccinations up to age seven months and up to age 18 months, that is, the later ones, a trend of treatment with antidepressants reducing the risk of non-vaccination was seen, and the results were very close to being statistically significant. It is documented in the literature that treatment of PPD with antidepressants has a positive effect on the mother's functioning (30, 31). Therefore, and based on our study results, it is likely that the medical treatment may have a positive impact on the rate of regular vaccination among children whose mothers had PPD. The conjectured reason for the absence of statistical significance is the sample size (*N* = 709), which was too small for this purpose. Further studies are needed in order to elucidate the effect of antidepressant treatment in PPD on vaccination rates of children.

On the other hand, the effects in the analysis of the group that received medical treatment in comparison with those who did not, involving vaccinations at age 1 month and age 2 months, that is, those that are given the shortest time after the birth, did not show significant differences between the group of mothers that received treatment and the group that did not, and no trend was observed. It is important to note that we did not examine when, after the EPDS questionnaire was filled out, the medical treatment actually began. It is possible that it began several months after the birth, and that no impact was therefore seen in the effects involving the first 2 months after the birth. Furthermore, it is important to keep in mind that most antidepressants begin to exert an effect after approximately 13 days and that full effectiveness is reached only after approximately 19 days (32). In other words, even if the medical treatment was begun immediately after the EPDS questionnaire was filled out, there is an additional lag time, so it is likely that no difference between the groups will be observed in the effects immediately following the birth. These results highlight the importance of monitoring PPD symptoms and the subsequent treatment for PPD.

## Limitations

Only 709 from 2,410 women with depression symptoms and 681 from 39,137 women without depression symptoms were matched, likely to induce a significant selection bias. After omitting from the 41,547 women the subjects with missing data, the children of the mothers in both groups were matched according to five variables. A matching was used to prevent confounding of the risk ratio. The socioeconomic index was the most restrictive variable for matching. Each residential area in the country, at the level of municipalities and local councils, is characterized by a different number (according to the Central Bureau of Statistics' geographic characterization code). Since, as far as we know, area-level socio-demographic factors may have an influence on vaccination rates (33), we decided to include this parameter in the matching criteria and not to make a covariate adjustment. It is common practice to adjust for as many variables as possible in observational studies in the hopes of reducing confounding by other variables. However, we were concerned that indiscriminate adjustment for variables using standard regression models may lead to biased estimates (34). The mothers needed to match all the variables. In that way, we tried to create two groups as similar as possible, but it came up in getting small sample sizes, and the individual analyses may lack power. Perhaps a matching 1:2 or 1:3 or using other analytical methods such as conditional regression and covariate adjustment would have been better and help to gain power. Regarding the low number of subjects born between 2006 and 2014 that we included, we believe that the main reason there were few subjects in that period is that the Ministry of Health of Israel issued in 2014 a procedure for locating women at risk for depression during pregnancy and childbirth and instructed the Health Maintenance Organizations (HMOs) to pass the EPDS questionnaire since then.

The characteristics of the matched and unmatched population were not compared. Since only 709 from 2410 women with depression symptoms and 681 from 39,137 women without depression symptoms were matched this comparison could be essential in order to rule out any protentional selection bias.

No matching was conducted according to the gestation age and the number of children in the family, which could be confounding factors. Mothers with two or more children find it more difficult to make sure that their children receive the regular vaccinations than mothers with only one child (11). In addition, it is possible that the gestation age, especially if the birth was full-term or premature, is a parameter that is likely to influence the parents' decision to have their children vaccinated or is likely to cause postponement of the vaccinations at the doctor's recommendation. Another possible confounding factor is the anti-vaccination attitude, which may have changed since 2006. We conducted a matching in which two of the variables for matching were the child's year of birth and socioeconomic index, with the intention to overcome this confounder. Family support may be considered as another confounder that wasn't addressed in the study.

By relying exclusively on the EPDS questionnaires in order to detect women with PPD symptoms, we may have omitted women with severe symptoms of postpartum depression who did not come to the mother and child clinics or did not fill out the questionnaire at all. Such women could include some with severe depression who find it difficult to leave home in order to continue their daily lives, or women hospitalized for psychiatric problems, for example.

The Ministry of Health's procedure distinguishes between three risk groups: low-risk (a score of <10 on the questionnaire), medium-risk (a score in the 10–12 range), and high-risk (a score of 13 or higher). It is possible that setting the EPDS threshold score at 10, above which the mother is assigned to the group “with PPD symptoms” or below which she is assigned to the group “without PPD symptoms,” resulted in the inclusion of many patients in the group” “with PPD symptoms” when they, in fact, were merely suffering from low spirits. This could have caused overestimation. It is possible that the inclusion in the PPD group in the study of patients who do not really suffer from PPD affected the results by giving us two groups that were more similar to each other than we would have obtained, had we included in the PPD group only patients with a score above 13. Our decision regarding the threshold score is in line with further studies on the subject (29, 35). Furthermore, According to the Ministry of Health, the use of a threshold score of 10 in order to detect PPD has attained worldwide acceptance, and we have therefore adhered to these guidelines in this study (36).

We did not include information about the date on which the medical treatment was begun after the questionnaire was filled out, and about the duration of the medical treatment. It is possible, for example, that an isolated dispensing of an antidepressant to a mother was included, and that this, therefore, does not necessarily indicate that the mother was treated with an antidepressant.

Regarding the sub-analysis comparing mothers with and without antidepressants, perhaps the mothers who received treatment with antidepressants have a more severe degree of depression and receive more support from the father and their families in caring for the children, which wasn't considered in the design of the study.

## Conclusion

In summary, this retrospective cohort study shows that PPD in a mother is not associated with a decline in the vaccination rates for routine vaccines. At the same time, the study showed that providing treatment with antidepressants for mothers in whom PPD has been detected tends to provide a protective effect against failure to give children regular vaccinations. An additional study is needed that will focus on the subject of treatment with antidepressants for women in whom PPD has been detected, and on an examination of the effect of the treatment on the vaccination rates of their children.

## Data Availability Statement

The data analyzed in this study is subject to the following licenses/restrictions: The dataset was provided by Kahn-Sagol-Maccabi Research and Innovation Institute, Maccabi Healthcare Services, Tel Aviv, Israel. Due to medical confidentiality, only encrypted information was provided. Requests to access these datasets should be directed to plotnik_l@mac.org.il.

## Author Contributions

ZG conceptualized, designed the study, methodology, reviewed, revised the manuscript, and approved the final manuscript as submitted. AZ revised the design and methodology, carried out the analyses, drafted the initial manuscript, and approved the final manuscript as submitted. GK gave scientific consultation during the design and the analyses of the study, reviewed the manuscript, and approved the final manuscript as submitted. GC revised the design and methodology, reviewed the manuscript, and approved the final manuscript as submitted. All authors contributed to the article and approved the submitted version.

## Funding

This work was funded by Marom, a research program for physicians and residents in Maccabi Healthcare Services, Tel Aviv, Israel and by Kahn-Sagol-Maccabi Research and Innovation Institute, Maccabi Healthcare Services, Tel Aviv, Israel.

## Conflict of Interest

The authors declare that the research was conducted in the absence of any commercial or financial relationships that could be construed as a potential conflict of interest.

## Publisher's Note

All claims expressed in this article are solely those of the authors and do not necessarily represent those of their affiliated organizations, or those of the publisher, the editors and the reviewers. Any product that may be evaluated in this article, or claim that may be made by its manufacturer, is not guaranteed or endorsed by the publisher.

## Abbreviations

CI: Confidence Interval
DTaP: Diphtheria-Tetanus-Acellular Pertussis Vaccine pediatric
DTP: Diphtheria-Tetanus-Pertussis
EPDS: Edinburgh Postnatal Depression Scale
HBV: Hepatitis B Vaccine
Hib: Haemophilus influenzae b Vaccine
IPV: Inactivated Polio Vaccine
MMR: Measles-Mumps-Rubella Vaccine
PPD: Post-Partum Depression
RR: Relative Risk.
