# Supplementary material for: No Association Between Maternal Post-partum Depression and Vaccination Uptake of Infants: A Matched Cohort Study in a Large Health Maintenance Organization Database in Israel
Source: Front Pediatr. 2022 Feb 8;9:771089. doi: 10.3389/fped.2021.771089 (PMC8860966; doi:10.3389/fped.2021.771089)
Supplement: Supplementary file 2 [file Data_Sheet_2.docx]

**Appendices**

Table 1. Routine Immunization Schedule 2019, as published on the Ministry of Health Website (37)

| **Age** | | | | | | | | | | | | | **Vaccinations** |
| --- | --- | --- | --- | --- | --- | --- | --- | --- | --- | --- | --- | --- | --- |
| **School** | | | | | **3^rd^ Year** | **2^nd^ Year** | | **1^st^ Year** | | | | |  |
| **13 Years (8^th^ Grade)** | **9 Years (4^th^ Grade)** | **8 Years (3^rd^ Grade)** | **7 Years (2^nd^ Grade)** | **6 Years (1^st^ Grade)** | **24 Months** | **18 Months** | **12 Months** | **6 Months** | **4 Months** | **2 Months** | **1 Month** | **Day of Birth** |  |
|  |  |  |  |  |  |  |  | HBV |  |  | HBV | HBV | Hepatitis B |
|  |  |  | IPV |  |  |  | IPV | IPV | IPV | IPV |  |  | Polio |
|  |  |  |  |  |  | BOPV |  | BOPV |  |  |  |  |  |
| Tdap |  |  | Tdap |  |  |  | DTAP | DTAP | DTAP | DTAP |  |  | Diptheria-Tetanus-Pertussis |
|  |  |  |  |  |  |  | Hib | Hib | Hib | Hib |  |  | Haemophilus Influenzae B |
|  |  |  |  |  |  |  | PCV13 |  | PCV13 | PCV13 |  |  | Pneumococcal Conjugate |
|  |  |  |  |  |  |  |  | Rota | Rota | Rota |  |  | Rotavirus |
|  |  |  |  | MMR |  |  | MMR |  |  |  |  |  | Measles-Mumps-Rubella |
|  |  |  |  | Var |  |  | Var |  |  |  |  |  | Varicella |
|  |  |  |  |  | HAV | HAV |  |  |  |  |  |  | Hepatitis A |
| HPV* |  |  |  |  |  |  |  |  |  |  |  |  | Human Papillomavirus |
|  | Flu | Flu | Flu |  |  |  |  |  |  |  |  |  | Influenza |

Notes: The IPV, Tdap, DtaP, Hib, MMR, and Varicella vaccines may be given in various combinations, as are available in Israel at that time.

bOPV, Bivalent Oral Polio Vaccine

*Will be given in 2 doses.

Table 2. EPDS questionnaire (8)

**Questionnaire for pregnant and postpartum women**

| **6. In the past 7 days, Things have been getting on top of me**  ___ Yes, most of the time I haven’t been able to cope at all  ___ Yes, sometimes I haven’t been coping as well as usual  ___ No, most of the time I have coped quite well  ___ No, have been coping as well as ever | **1. In the past 7 days, I have been able to laugh and see the funny side of things**  ___ As much as I always could  ___ Not quite so much now  ___ Definitely not so much now  ___ Not at all |
| --- | --- |
| **7. In the past 7 days, I have been so unhappy that I have had difficulty sleeping**  ___ Yes, most of the time  ___ Yes, sometimes  ___ Not very often  ___ No, not at all | **2. In the past 7 days, I have looked forward with enjoyment to things**  ___ As much as I ever did  ___ Rather less than I used to  ___ Definitely less than I used to  ___ Hardly at all |
| **8. In the past 7 days, I have felt sad or miserable**  ___ Yes, most of the time  ___ Yes, quite often  ___ Not very often  ___ No, not at all | **3. In the past 7 days, I have blamed myself unnecessarily when things went wrong**  ___ Yes, most of the time  ___ Yes, some of the time  ___ Not very often  ___ No, never |
| **9. In the past 7 days, I have been so unhappy that I have been crying**  ___ Yes, most of the time  ___ Yes, quite often  ___ Only occasionally  ___ No, never | **4. In the past 7 days, I have been anxious or worried for no good reason**  ___ No, not at all  ___ Hardly ever  ___ Yes, sometimes  ___ Yes, very often |
| **10. In the past 7 days, The thought of harming myself has occurred to me** ___ Yes, quite often  ___ Sometimes  ___ Hardly ever  ___ Never | **5. In the past 7 days, I have felt scared or panicky for no very good reason**  ___ Yes, quite a lot ___ Yes, sometimes ___ No, not much ___ No, not at all |

**Comments by the woman:**

**_____________________________________________________________________ _____________________________________________________________________**

**Please list the overall score here:** |____|____|

______________ _____________ _____________ _____________

**woman's name nurse's name station city**

**Date of completion of the questionnaire:** __________________

Table 3. List of antidepressants included in the study

| **others** | **TeCAs** | **TCAs** | **SNRIs** | **SSRIs** |
| --- | --- | --- | --- | --- |
| vortioxetine | mianserin | clomipramine | venlafaxine | citalopram |
| bupropion | maprotiline | desipramine | duloxetine | fluvoxamine |
| mirtazapine |  | amitriptyline | milnacipran | fluoxetine |
| esketamine |  | doxepin | desvenlafaxine | paroxetine |
| hypericum |  | opipramol |  | sertraline |
| phenelzine |  | imipramine |  | escitalopram |
| nialamide |  | trimipramine |  |  |
| trazodone |  | dibenzepin |  |  |
| moclobemide |  | nortriptyline |  |  |
| reboxetine |  |  |  |  |
| tranylcypromine |  |  |  |  |
| nefazodone |  |  |  |  |

SNRIs, Serotonin-Norepinephrine Reuptake Inhibitors

SSRIs, Selective Serotonin Reuptake Inhibitors

TCAs, Tricyclic antidepressants

TeCAs, Tetracyclic antidepressants
